# Variability in the Duration of Designated Pediatric Orthopaedic Rotations Among US Residency Programs

**DOI:** 10.5435/JAAOSGlobal-D-20-00160

**Published:** 2021-01-19

**Authors:** Bensen Fan, Caixia Zhao, Sanjeev Sabharwal

**Affiliations:** From the Atlantic Medical Group, Pediatric Orthopaedic Surgery, Morristown, New Jersey (Dr. Fan); Rutgers- New Jersey Medical School, Newark, New Jersey (Dr. Zhao); and Department of Orthopedics, University of California, San Francisco, UCSF Benioff Children's Hospital of Oakland, Oakland, California (Dr. Sabharwal).

## Abstract

Our goal was to assess the variability in the assigned duration of pediatric orthopaedic rotation among US allopathic orthopaedic residency programs to see how pediatrics is incorporated into surgical education.

**Methods:**

Using publicly available information for US allopathic orthopaedic residency programs in 2019, we retrospectively collected data on the assigned duration of pediatric orthopaedic rotation and variables such as number and sex of residents, number of orthopaedic faculty, university- versus community-based programs, outsourcing residents to unaffiliated hospital for pediatric exposure, specialty of program leadership, and presence of pediatric orthopaedic fellowship in the home program.

**Results:**

One hundred thirty-eight of the 146 (95%) eligible allopathic orthopaedic residency programs provided sufficient information. The average time assigned to a pediatric rotation during residency was 6 months (range: 2 to 11 months). Overall, 43/146 (29%) programs outsourced their pediatric training to another institution. A correlation was noted between the length of pediatric rotation and percentage of pediatric orthopaedic faculty (*P* = 0.0007, r = 0.3).

**Conclusions:**

The impact of the variability in the duration of duration of pediatric orthopaedic rotation on the clinical knowledge and skills acquired by the resident during training needs further study.

The American Board of Orthopaedic Surgeons (ABOS) requires a minimum of 6 months of pediatric orthopaedic training, and this clinical experience can vary among residency programs.^1^ Pediatric orthopaedics and trauma are the only two orthopaedic subspecialties that have been designated with a minimum number of months of required clinical exposure during residency, whereas other subspecialties, such as foot and ankle, do not have an ABOS-mandated minimum duration required during residency training.^2^

Our goal was to study the variability of pediatric rotation schedule among US allopathic orthopaedic residency programs. In addition, we wanted to examine any potential relationships between the duration of assigned pediatric orthopaedic rotation and program-specific characteristics such as the number of residents and faculty, subspecialty of faculty leadership, University affiliation, outsourcing of pediatric orthopaedic rotation, and presence of a pediatric orthopaedic fellowship affiliated with the residency program. We hope that our findings of this pilot study will serve as a foundation for futures studies in orthopaedics and other surgical specialties to examine the factors affecting the pediatric subspecialization training as a resident.

## Methods

No IRB approval was necessary because this study was based on publicly available information. A funding source was not needed for this investigation. We did an extensive search of the websites of 187 orthopaedic residency programs as listed on Electronic Residency Application Service (ERAS) (https://services.aamc.org/eras/erasstats/par/display.cfm?NAV_ROW=PAR&SPEC_CD=260) in December 2019. Using the Wayback Machine (https://archive.org/web/), we tracked whether the website was changed within the previous year. Residency programs were excluded if they were osteopathic programs. New residency programs with less than 5 years of existence were also excluded because they do not have a full class of residents and have an incomplete rotation schedule.

Demographic variables searched for each allopathic orthopaedic residency program included the total number of residents in the program, number of female residents, number of full-time faculty, the specialty of the department chairman and program director, university-based versus community-based program, number of months spent on pediatric orthopaedics, outsourcing residents to unaffiliated hospital for training, and whether pediatric orthopaedic fellowship was offered in the home program.

A university-based residency program was defined as the one in which the main teaching hospital for postgraduate orthopaedic training was the primary teaching hospital for a medical school. A community-based program, on the other hand, was the one in which the main teaching hospital was not the primary teaching hospital for a medical school, even if some academic affiliation was present. The duration of assigned clinical rotations was recorded in months. Any schedules listed in “weeks” was converted to “months”. If rotation schedules spanned over two consecutive years of training, then the number of months was split evenly between the 2 years (eg, 4 months of pediatrics during PG2 and PG3 = 2 months PG2, 2 months PG3). Programs that did not specify their rotations were designated as unspecified and excluded from the final analysis. If data obtained from the website were incomplete, residency program coordinators were e-mailed to obtain the missing information. If there was no response by two e-mail reminders, a phone call was made to the residency program coordinator to try to obtain the missing information. Data were organized into range and average number of months spent on each rotation.

Using SAS, version 9.3 software (SAS Institute Inc, Cary, North Carolina), statistical analysis was did to determine relationships between certain continuous and categorical variables. The student t-test was used to compare means with normalized data (such as number of residents, female residents, and months spent on rotation), and chi-square analysis was used to compare categorical data (such as presence of a pediatric orthopaedic fellowship in the home program, US census region, and university-based versus community-based-based program). Analysis of Variance (ANOVA) was used to compare differences between means of three or more independent groups and time allotted to pediatric rotation. Pearson correlation coefficient was used to determine the strength of a linear relationship among select variables and the duration of pediatric orthopaedic rotation. Differences were considered significant at *P* < 0.05.

## Results

### Program Demographics

One hundred and eighty-seven orthopaedic residency programs (allopathic and osteopathic programs participating in the match) were listed on the ERAS website. One hundred and forty-six (146) programs met our inclusion criteria (37 programs were osteopathic programs, four new allopathic programs with less than a full 5 year class of residents). Of the 146 program websites, 115 university-based and 31 community-based programs were present (Table 1). An average of five pediatric orthopaedic faculty (range 0 to 37) were present per residency program. Although 37 pediatric orthopaedic faculty in a single program was an outlier, residency programs affiliated with a stand-alone pediatric hospital or a combined hospital system tended to have a higher number of pediatric orthopaedic faculty. To account for this variability in size of programs, we focused on the percentage of pediatric faculty in the orthopaedic department rather than the absolute number of pediatric faculty. Five orthopaedic department chairman (5/146; 3%) and 13 program directors (13/146; 9%) specialized in pediatric orthopaedics.

**Table 1:** Demographic Results Summary Comparing University-Based and Community-Based Programs

	All Programs (n = 146)	University Programs (n = 115)	Community Programs (n = 31)	University versus Community, *P* value
Length of pediatric rotation^a^ (mo), mean (range)	6.0 (1.5-10.5)	6.1 (1.5-10.5)	5.8 (4-7)	0.30
Number of female residents, mean (range)	4 (0-16)	4 (0-16)	2 (0-7)	0.0001
Percent of pediatric faculty, mean (range)	14% (0-61)	16% (0-61)	6% (0-27)	0.0001
Pediatric chairman, number (%)	5/146 (3%)	5/115 (4%)	0/31 (0%)	0.59
Pediatric program director, number (%)	13/146 (9%)	13/115 (11%)	0/31 (0%)	0.04
Outsource pediatric rotation, number (%)	43/146 (29%)	21/115 (18%)	22/31 (71%)	0.0001
Programs offer a pediatric fellowship, number (%)	43/146 (29%)	41/115 (36%)	2/31 (6%)	0.002

a Of 146 programs, four programs excluded for mentorship model and four programs did not supply rotation data by e-mail or phone.

Thirty-nine programs were in the Northeast, 36 in the Midwest 50 in the South, and 21 in the West. Forty-three programs also offered a pediatric fellowship at their home institution. A higher average percentage of pediatric orthopaedic faculty was noted at university-based programs compared with community programs (16%, versus 6% *P* = 0.03). We also noted a higher average number of female residents at university-based programs compared with community programs (mean # female residents 4 versus 2, *P* = 0.0001) (Table 1).

Using our data acquisition protocol, we found that 21 programs did not specify their pediatric rotation schedule on their websites. We were able to obtain the rotation schedule from 10 more programs via e-mail and seven more programs via telephone. We were unsuccessful to obtain rotation schedule for the remaining four programs—three programs did not answer and one program refused to answer because they were undergoing a clinical competency review. Four other programs used a mentorship model curriculum rather than service-based curriculum and so could not provide complete data regarding the duration of pediatric orthopaedic rotation. Thus, we obtained complete information regarding the duration of pediatric orthopaedic rotation for 138 of the 146 (95%) orthopaedic residency programs (Table 1).

### Duration of Pediatric Orthopaedic Rotations

The average time spent on an assigned pediatric orthopaedic rotation during the entire residency was 6 months (range: 2 to 11 months). Forty (40/138; 29%) programs offered less than 6 months of assigned pediatric orthopaedic rotation. The most common training year for a pediatric rotation was during PGY 4, followed by PGY 3, although on average PGY 3 rotations were longer (Table 2).

**Table 2:** Comparison of Orthopaedic Residency Program Demographics and Months Spent on Pediatric Orthopaedic Rotation

	# Programs	Average # months	*P*
Total	138^a^	6.0	n/a
Pediatric rotation training year			
PG1	36	0.36	n/a
PG2	80	1.39	n/a
PG3	88	1.80	n/a
PG4	90	1.68	n/a
PG5	50	0.81	n/a
Academic programs//Community programs	110//28	6.1//5.8	0.3
Outsource//No outsource	43//95	5.7//6.2	0.05
With fellowship//Without fellowship	43//95	6.2//6.0	0.42

a Of 146 programs, four programs were excluded for mentorship model and four other programs did not supply the duration of pediatric rotation data.

A positive correlation existed between the length of pediatric rotation and percentage of pediatric faculty (*P* = 0.0007, r = 0.3). No association existed between having a chairman or residency program director specializing in pediatric orthopaedics and the time allocated for the pediatric orthopaedic rotation (*P* = 0.2). No correlation was noted between variables such as the size of the program (total number of residents), having outsourced pediatric rotations, or the presence of a pediatric orthopaedic fellowship on the duration of pediatric orthopaedic rotation (Table 1).

### University- Versus Community-Based Programs

Although the assigned duration of pediatric rotation was similar for university- versus community-based programs (*P* = 0.3) (Table 2), a greater percentage of community-based programs (22/31, 71%) outsourced their pediatric training (*P* < 0.0001) compared with university-based programs (21/115, 18%) (Table 1). Of note, 100% (n = 22) of the community-based programs that outsourced their pediatric training sent their residents to a university-based children's hospital. Ten of these programs sent their residents to a Shiner's hospital. Overall, 43/146 (29%) programs outsourced their pediatric training to another institution.

## Discussion

Based on our review of the literature, this is the first study assessing the variability in pediatric orthopaedic rotations among allopathic orthopaedic residency programs in the United States with over 95% complete data. Previous studies on the overall orthopaedic rotations were based on a lower number of programs.^3,4^ We found substantial variability in the duration and format of pediatric orthopaedic rotations among US orthopaedic residency programs. Not all US allopathic orthopaedic residency programs (n = 40/138, 29%) met the minimum ABOS requirement of 6 months of pediatric orthopaedics and at least 43/146 (29%) of orthopaedic residency programs (Table 1) supplement their residents' pediatric experience by arranging to have their residents spend time at a pediatric institution that is not primarily affiliated with the home program. Of note, rotations exist other than pediatric orthopaedics that may include pediatric experience. For example, general, oncology, and trauma rotations often include pediatric orthopaedic patients. However, pediatric orthopaedic principles covered during a dedicated pediatric rotation are often diluted by the mix of adult and pediatric patients.

Historically, general orthopaedic surgeons did both adult and pediatric fracture care; however, nationally, an increasing trend exists for fractures in children to be managed by pediatric orthopaedic specialists.^5-7^ Fewer orthopaedic surgeons consider themselves as generalists.^5,8^ We feel that orthopaedic resident education should provide enough clinical experience (currently 6 months) for the graduating resident to recognize and manage urgent and emergent pediatric orthopaedic emergencies including surgical management of open fractures, compartment syndrome, and septic arthritis especially when a pediatric orthopaedic surgeon is unavailable.^9-11^

The pediatric curriculum is critical to orthopaedic resident education and training as reflected in the content of the Orthopaedic In-Training Exam (OITE), with 13% of questions involving a pediatric topic, second only to the number of trauma questions.^12^ Recently, Franklin et al^13^ noted that weekly didactic conference in pediatric orthopaedics improved in-training examination scores in the pediatric section. Although number of OITE questions do not necessarily reflect value in orthopaedic practice, these tests provide a standardization of knowledge acquisition that should not be simply ignored. The relevance of pediatric orthopaedic training in clinical practice, in-training examinations, and Part 1 of ABOS board examination^12^ and the evolving ACGME guidelines and US healthcare structure and manpower led us to inquire into the variability of pediatric orthopaedic rotations among residency programs.

### Changing Healthcare, Training Requirements, and Workforce

Recent workforce projections suggest that the volume of pediatric orthopaedic patients has remained relatively stable from 2000 to 2014.^14^ Based on the Kids Inpatient Database (KID), 60,008 pediatric orthopaedic–related discharges existed in 1997 versus 65,481 pediatric orthopaedic–related discharges in 2012.^14^ The most common orthopaedic diagnosis were adolescent idiopathic scoliosis, supracondylar humerus fracture, and femoral shaft fracture.^14^ Based on the current ACGME guidelines, residents need to log a minimum of five pediatric elbow trauma cases (supracondylar or epicondylar or condylar humerus fracture fixation) and 200 procedures involving patients younger than 18 years old.

### Variability Among Programs

Despite changes to resident curriculum since 2003, no clear evidence exists of any substantial impact regarding resident education.^15^ In fact, OITE scores remained unchanged from 2003 to 2008 (after ACGME mandated work hour restrictions), compared with 2000 to 2003.^15,16^ Some authors have actually noted an increase in case volume among residents despite implementation of work-hour restrictions.^15^ As trends shift toward patient safety, residency programs have begun to develop simulation-based education.^17-20^

As orthopaedic education changes with the times, we devised this study as an initial foray into analyzing pediatric rotations so that we have a basis for future studies detailing case volume and competencies. Through our data collection, we observed wide variability in the size and demographics of the residency programs, including percentage of pediatric orthopaedic faculty and assigned duration of pediatric orthopaedic rotations. Despite these differences, most residency programs are finding creative ways to meet the minimum requirements for adequate training. The fact that almost one-third of all residency programs partner with nonaffiliated pediatric institutions is a testament to their commitment to a well-rounded resident training, and such collaborative efforts should be recognized.

### Study Limitations

One obvious drawback was that our analysis was largely based on available online information on the individual orthopaedic residency programs website. The retrieved data represent a snapshot in time, ie, in December 2019, when the data were collected and may not be reflective of the evolving educational curriculums that are ubiquitous. The publicly available data regarding pediatric orthopaedic experience during residency are limited. In addition to case log data being unavailable to programs, variability exists in reporting of the data. Despite this, our study is the most complete of its kind, with observations based on data collected on over 95% of US allopathic orthopaedic residency programs.

Another limitation of our study was the inability to access case logs and correlate surgical exposure with the duration of pediatric orthopaedic rotation. It is quite plausible that the designated time on rotations may not reflect the actual number of pediatric cases done by the resident and their level of involvement, or the level of clinical competency achieved at the end of the pediatric rotation. In addition, during some clinical rotations, such as night float, oncology, and upper extremity, the resident may encounter both pediatric and adult patients. This would allow for unaccounted pediatric experience that were not captured based on our methodology. An analysis of case logs may have revealed a different conclusion regarding pediatric orthopaedic experience during residency training. However, substantial variability exists in case logging practices among trainees.^21^

We hope that our findings can serve as an impetus for programs to standardize pediatric orthopaedic education during residency training. We found a substantial variability in the designated duration of pediatric rotations; however, we do not know whether this variability in the duration of assigned rotation correlates with clinical competence. It is plausible that the longer rotations may have a more dilute case load, whereas compact rotations can still have a higher frequency of cases and more meaningful education.

We hope that this study will serve as a baseline for educational leaders to examine the content and curriculum of pediatric orthopaedic training in more detail, including the impact of variables such as resident case log volume, faculty-resident ratios, and rotations in subspecialized units versus combined rotations covering two or more subspecialties on the degree of cognitive and surgical competency gained by the trainee.

## Conclusion

In summary, we found substantial variability in the structure and assigned duration of pediatric orthopaedic rotations among US orthopaedic residency programs. Not all US allopathic orthopaedic residency programs (40/138; 29%) met the minimum ACGME requirement of 6 months of pediatric orthopaedics, and at least 43/146 (29%) of orthopaedic residency programs supplement their residents' pediatric experience by making arrangements to have their residents spend time at a pediatric institution that is not primarily affiliated with the home program. Despite differences in pediatric curriculums across residency programs, residents may still meet minimum case requirements. We were also unable to ascertain the educational impact, if any, of having residents rotate at nonprimary-affiliated pediatric hospitals. However, it is encouraging to see cooperative efforts between institutions of different sizes and composition to provide adequate experience in pediatric orthopaedics, with the goal of providing a well-rounded musculoskeletal education and training.
